# MRI Findings of Uterine Adenofibroma With Squamous Metaplasia

**DOI:** 10.7759/cureus.106175

**Published:** 2026-03-31

**Authors:** Akane Kaizu, Iichiro Osawa, Kaiji Inoue, Kosei Hasegawa, Masanori Yasuda

**Affiliations:** 1 Radiology, Saitama Medical University Hospital, Moroyama, JPN; 2 Gynecologic Oncology, Saitama Medical University International Medical Center, Hidaka, JPN; 3 Pathology, Saitama Medical University International Medical Center, Hidaka, JPN

**Keywords:** mri, papillary projections, squamous metaplasia, uterine adenofibroma, uterus

## Abstract

Uterine adenofibroma is an extremely rare, benign, mixed epithelial and mesenchymal tumor, and its imaging characteristics remain poorly characterized because only a small number of cases have been reported. Accurate preoperative diagnosis can be difficult, as the clinical and radiological features of uterine adenofibroma often overlap with those of endometrial carcinoma, adenosarcoma, and other lesions of the endometrial cavity. Very few studies have documented detailed MRI findings, and further case reports are needed to establish distinctive imaging patterns.

We report the case of an 84-year-old woman who presented with genital bleeding and a uterine mass on imaging. MRI revealed a mainly solid tumor with small cystic components occupying the endometrial cavity. Dynamic contrast-enhanced MRI demonstrated linear surface enhancement in the early phase and papillary projections with gradually increasing enhancement in the delayed phase. These imaging findings were consistent with the histopathological features of the tumor, which showed surface papillations and widespread squamous metaplasia. The presence of prominent squamous metaplasia likely contributed to the marked and progressive surface enhancement, a feature that distinguished this case from previously reported adenofibromas.

This report highlights the importance of recognizing unique MRI characteristics, particularly papillary surface enhancement patterns, when evaluating endometrial cavity tumors. Further accumulation of imaging-pathology correlated reports may assist in improving the diagnostic accuracy of this rare tumor type.

## Introduction

Uterine adenofibroma is an exceedingly rare benign biphasic epithelial and mesenchymal neoplasm [1]. It most commonly occurs in older women but has been reported across a broad age range [2]. Macroscopically, it typically presents as a polypoid mass within the uterine cavity [3], and histologically, it is defined by surface papillations and cystic spaces. Preoperative diagnosis remains difficult because imaging findings often overlap with those of malignant endometrial tumors, including endometrial carcinoma and adenosarcoma. Moreover, MRI features of uterine adenofibroma remain poorly characterized due to the limited number of reported cases [4,5]. We report a case of uterine adenofibroma with marked squamous metaplasia demonstrating distinct papillary surface enhancement on dynamic MRI, with radiologic and pathologic correlation.

## Case presentation

**Figure 1:**
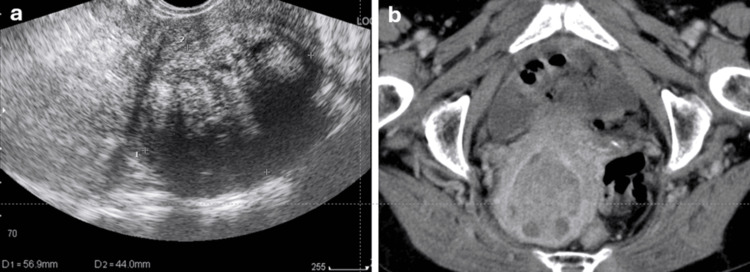
Preoperative imaging findings (a) Transvaginal ultrasonography shows an enlarged uterus with a well-defined echogenic mass occupying the endometrial cavity. The mass demonstrates heterogeneous internal echogenicity, consistent with a solid lesion containing small cystic components. (b) Contrast-enhanced axial computed tomography image demonstrates a well-circumscribed mass filling and expanding the endometrial cavity. The mass shows heterogeneous enhancement relative to the myometrium, with focal non-enhancing areas suggestive of cystic components. No fat density or calcification is observed

Our patient was an 84-year-old woman who presented with genital bleeding and a uterine mass identified on imaging. Among the tumor markers, carbohydrate antigen 19-9 (CA19-9) was elevated to 189 U/mL at the initial evaluation. On repeat testing before surgery, the level had decreased to 83 U/mL. This decline suggested a possible association with benign or inflammatory conditions rather than malignancy. A postoperative follow-up measurement of CA19-9 was not obtained. She reported no prior medical history of tamoxifen or oral contraceptive use. The patient was referred to our hospital from another hospital for further evaluation. Pelvic examination revealed a cystocele and ulceration of the vaginal wall, likely secondary to the pessary treatment. Transvaginal ultrasonography revealed an enlarged uterus with an echogenic mass in the endometrial cavity (Figure 1a). On non-contrast CT, the intrauterine mass was isodense relative to the myometrium. No fat density or calcification was observed in the mass. Contrast-enhanced CT revealed a well-defined and heterogeneously enhancing mass filling and expanding the endometrial cavity. Some non-enhancing lesions were observed within the mass, suggesting cystic components (Figure 1b).

**Figure 2:**
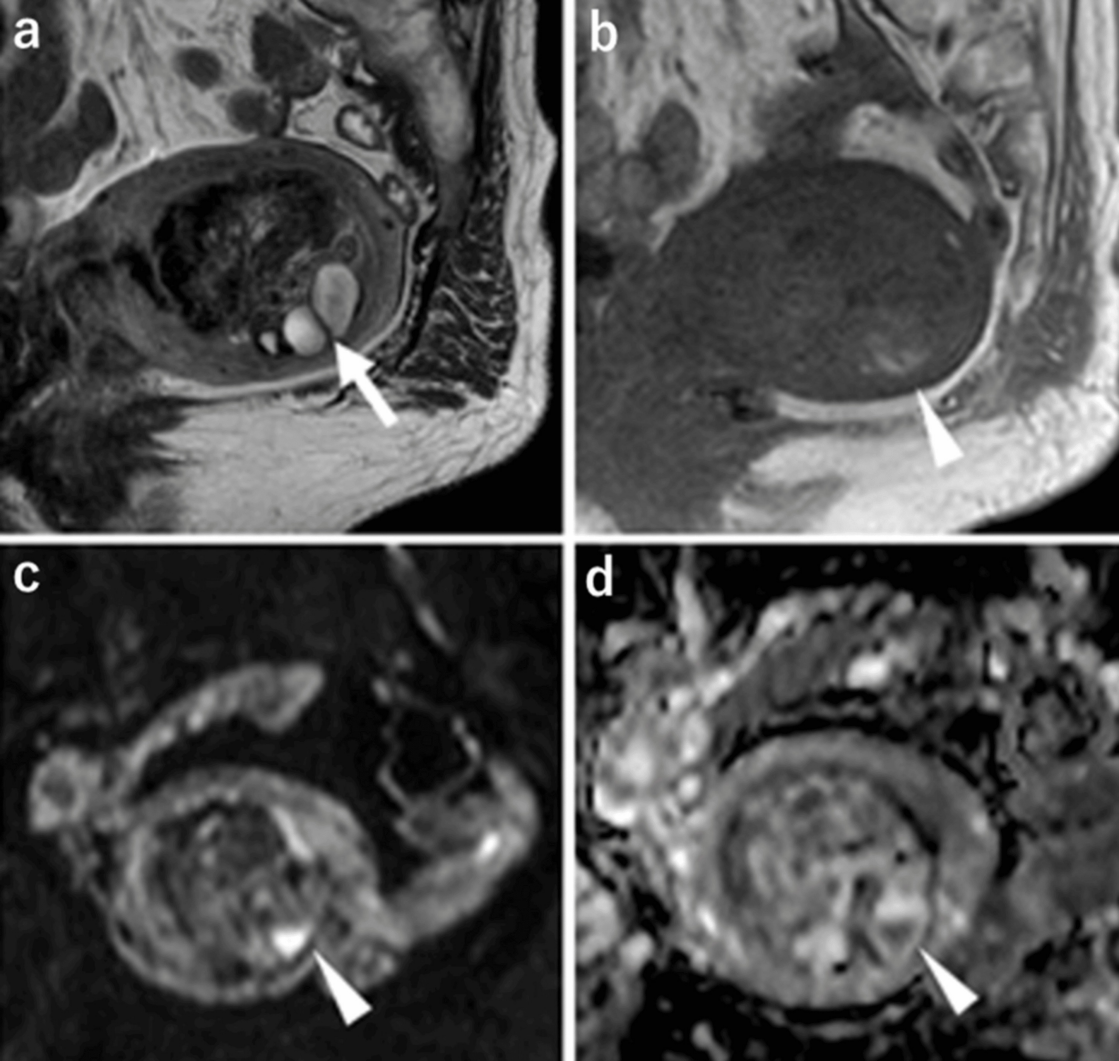
Pelvic MRI findings of the uterine mass (a) Sagittal T2-weighted image showing a mass occupying the uterine cavity, demonstrating heterogeneity and hypointensity relative to the myometrium, with multiple hyperintense foci indicative of cysts (arrow). (b) Sagittal T1-weighted image showing an isointense mass relative to the myometrium with small hyperintense foci suggestive of a hemorrhage (arrowhead). (c, d) Axial diffusion-weighted imaging with a b-value of 1000 s/mm^2^. (c) The solid components in the tumor demonstrate low signal intensity with high apparent diffusion coefficient values (d), except for the areas of hemorrhage (arrowhead). MRI: magnetic resonance imaging

The patient underwent pelvic MRI using a 3.0 Tesla MRI scanner (Achieva, Philips Medical Systems, Best, Netherlands). The MRI protocol is detailed in Table 1. On non-contrast MRI, a well-defined mass, measuring 52 × 48 × 39 mm, was identified in the uterine cavity. The mass showed heterogeneous hypointensity relative to the myometrium on turbo spin-echo T2-weighted imaging (T2WI) with multiple high-signal areas, indicative of small cysts (Figure 2a). Spin-echo T1-weighted imaging (T1WI) revealed an isointense mass relative to the myometrium with high-signal foci that appeared consistent with hemorrhage (Figure 2b). On diffusion-weighted imaging (DWI) with a b-value of 1,000 s/mm2, the solid components, excluding the hemorrhage, showed hypointensity relative to the myometrium and elevated apparent diffusion coefficient (ADC) values (Figures 2c, 2d).

**Table 1:** MRI - sequences and parameters MRI: magnetic resonance imaging; DWI: diffusion-weighted imaging; T2WI: T2-weighted imaging; Fs T1WI: fat-suppressed T1-weighted imaging; Fs-Gd T1WI: fat-suppressed gadolinium contrast-enhanced T1-weighted imaging; 3D-Fs-Gd T1WI: three-dimensional isotropic fat-suppressed gadolinium contrast-enhanced T1-weighted imaging

Sequence	DWI	T2WI	Fs T1WI	Dynamic Fs-Gd T1WI	3D-Fs-Gd T1WI
b value (s/mm^2^)	0, 500, 1000	-	-	-	-
Cross-section	Axial	Sagittal	Sagittal	Sagittal	Coronal
Repetition time (ms)	6750	5781	3	3–5	4
Echo time (ms)	75	95	2	2–3	2
Field of view (cm)	32 ×32	28 × 28	28 × 28	28 × 28	26 × 26
Slice thickness (mm)	4	4	4	4	0.9
Dynamic phase (s)	-	-	-	35, 65, 125	-

**Figure 3:**
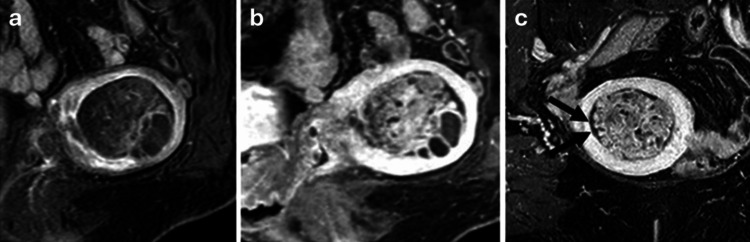
Contrast-enhanced MRI of the pelvis (a) In the early phase of the dynamic contrast-enhanced study (sagittal plane), the solid components of the mass exhibit heterogeneous enhancement. (b) In the delayed phase (sagittal plane), the mass shows prolonged enhancement. (c) The papillary surface of the mass is clearly visualized on thin-slice three-dimensional axially reformatted T1-weighted images after gadolinium administration (coronal plane, arrows). Panels (a) and (b) were selected to best demonstrate contrast enhancement patterns, whereas panel (c) was obtained from a different optimized slice and imaging plane to better depict papillary surface morphology; therefore, identical anatomical sections are not shown across panels MRI: magnetic resonance imaging

The mean ADC value of the solid component was 1.82 ×10⁻³ mm²/s (ROI 20 mm²), which was higher than that of the myometrium (1.20 ×10⁻³ mm²/s). Malignant tumors such as endometrial carcinoma typically demonstrate lower ADC values (approximately 0.8-1.2 ×10⁻³ mm²/s) due to high cellularity. On three-dimensional (3D) contrast-enhanced dynamic MRI, the mass demonstrated papillary projections and weak heterogeneous enhancement in the early phase (Figure 3a), with progressively increased surface enhancement in the delayed phase (Figure 3b). The enhancement was consistently hypointense compared to the myometrium; a 0.9 mm isovoxel 3D gradient-echo T1-weighted sequence after gadolinium enhancement revealed papillary projections (Figure 3c).

Although papillary surface enhancement and the absence of diffusion restriction were observed, uterine adenofibroma was not initially suspected because these findings have not been widely reported in the literature. Recognition of papillary surface architecture on thin-slice contrast-enhanced MRI may serve as a useful clue, prompting consideration of an adenofibroma in future cases. Differential diagnoses included atypical polypoid adenomyoma (APAM), partially cystic submucosal myoma, and adenosarcoma. Because endometrial cancer associated with endometrial polyps could not be ruled out, given the patient’s advanced age, total abdominal hysterectomy and bilateral oophorectomy were performed.

**Figure 4:**
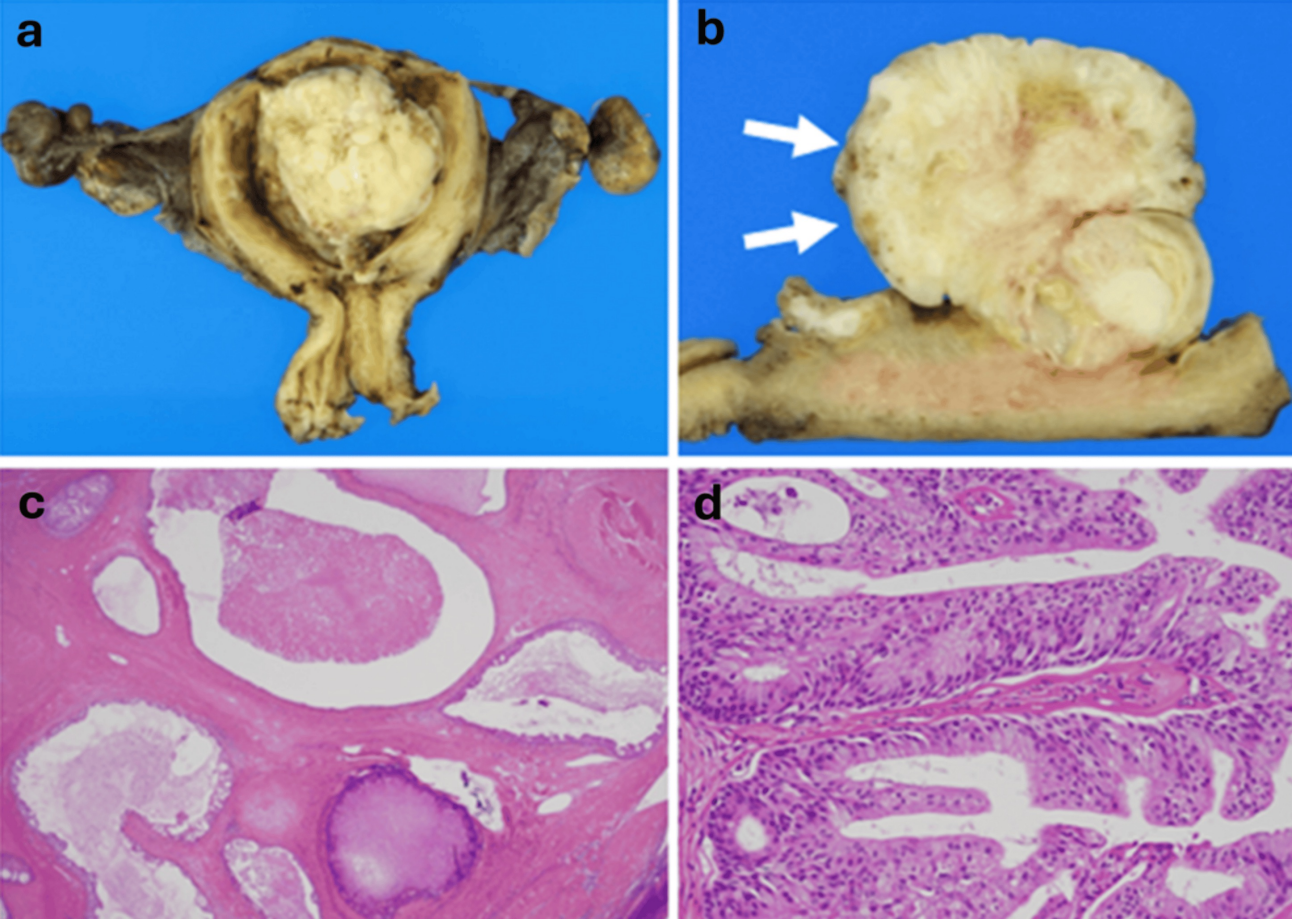
Pathology of the uterus (a, b) Macroscopic examination reveals a polypoid mass occupying the uterine lumen. (a) The mass contains papillary and cystic areas within the solid component (b, arrows) and papillations on the surface (b, arrowheads). (c, d) Microscopic examination using hematoxylin and eosin (H&E) staining - (c: low-power view (×4), d: high-power view (×20) - reveals that the mass is composed of epithelial and stromal components with surface papillations. The epithelial components are endometrial-type glands with diffuse squamous metaplasia. The stromal components surrounding the glands are fibroblastic and show no cytological atypia

Macroscopic examination revealed a polypoid mass, measuring 65 × 53 × 47 mm, in the endometrial cavity (Figure 4a). The incisional cross-sectional surface showed solid areas with cystic structures and papillary projections, as indicated by the arrows in Figure 4b. Microscopic examination revealed that the mass was composed of endometrial-type glands with squamous metaplasia and stroma, along with club-shaped papillations into the clefts (Figure 4c). The stroma was largely replaced by fibrous tissue (Figure 4d) and lacked smooth muscle components. Mitotic figures were not identified, and no malignant cells were observed. The constellation of histological features was consistent with adenofibroma. The patient was discharged uneventfully on postoperative day 10. More than five years later, there has been no evidence of recurrence.

## Discussion

Although uterine adenofibroma is generally considered a benign tumor, several reports have described the possibility of recurrence or misdiagnosis as adenosarcoma in cases of incomplete excision [2]. The potential for true malignant transformation remains a subject of debate. Adenofibromas have also been reported in different sites of the female genital tract, including the cervix, where they may present as polyp-like or papillary growths [1,6]. Histologically, uterine adenofibromas are characterized by papillary surface projections and club-shaped projections into cystic spaces, and approximately half of the reported cases show small cystic lesions [7]. Macroscopically, these tumors typically present as lobulated polypoid masses.

Konishi et al. described uterine adenofibromas as well-circumscribed intrauterine lesions with cystic areas and solid components showing low signal intensity on T2-weighted imaging and isointensity on T1-weighted imaging [8]. Hayasaka et al. reported comparable MRI signal features and enhancement patterns in adenofibroma associated with tamoxifen therapy [9]. In the present case, the tumor was also predominantly solid with minor cystic components, and the solid portion showed low signal intensity on T2WI and isointensity on T1WI, findings consistent with these prior reports.

Elsammak et al. demonstrated that malignant tumors such as endometrial carcinoma typically show reduced ADC values because of high cellularity and restricted water diffusion [10]. Agostinho et al. noted that submucosal leiomyomas may exhibit areas of increased signal intensity because of degenerative changes, whereas adenomyomas may show hyperintense regions due to the presence of dilated endometrial glands on MRI [11]. ADC values in the present case were measured by placing a circular region of interest within the solid component while carefully excluding cystic and hemorrhagic regions. Therefore, the relatively high ADC value observed in this case may further support the benign nature of the lesion. Marcus reported that squamous metaplasia can occur as a result of chronic inflammation, endometrial hyperplasia, and malignancy, conditions that may enhance vascular permeability and lead to irregular surface morphology [12].

Histopathological examination in the present case demonstrated inflammatory changes in the background endometrium, including surface erosion and infiltration by inflammatory cells. Chronic inflammation associated with pessary treatment may therefore have played a role in the development of squamous metaplasia, although a causal relationship cannot be definitively established. Yan et al. reviewed inflammatory mechanisms in chronic endometritis and emphasized that persistent inflammation is a well-established factor linked to epithelial metaplastic changes in the endometrium [13].

Kaji et al. reported a uterine adenofibroma mimicking endometrial carcinoma that appeared as a polypoid intrauterine mass with heterogeneous signal intensity and mild enhancement on MRI [14]. Lee et al. described CT and MR findings of uterine adenofibroma and adenosarcoma, reporting mixed signal intensity with cystic components and mild-to-moderate contrast enhancement [15]. Watanabe et al. also reported a well-defined polypoid intrauterine lesion with cystic structures and relatively homogeneous enhancement on MRI [4]. A comparison of MRI findings from these previously reported cases and the present case is summarized in Table 2.

**Table 2:** Comparison of MRI findings between previously reported uterine adenofibroma cases and the present case MRI: magnetic resonance imaging; DWI: diffusion-weighted imaging; T2WI: T2-weighted imaging

Study	Patient age	MRI morphology	Signal characteristics	Contrast enhancement
Kaji et al. [14]	63 years	Polypoid intrauterine mass	Heterogeneous on T2WI with small cystic areas	Mild enhancement
Lee et al. [15]	55 years	Endometrial cavity mass	Mixed signal intensity with cystic components	Mild to moderate enhancement
Watanabe et al. [4]	75 years	Well-defined polypoid lesion	High signal areas on T2WI suggesting cystic structures	Relatively homogeneous enhancement
Present case	84 years	Predominantly solid mass with papillary surface	Small cystic components within the tumor	Early linear surface enhancement and progressively enhancing papillary projections

When evaluating T2-hypointense masses in the endometrial cavity, differential diagnoses include submucosal leiomyomas, adenomyomas, APAM, endometrial polyps, endometrial carcinoma, and adenosarcomas. A distinguishing feature of uterine adenofibromas is the presence of club-shaped papillations, which can be more distinctly visualized on thin-slice three-dimensional gradient-echo T1-weighted images after contrast enhancement [8].

Although surface papillations are well recognized histologically in uterine adenofibromas, radiologic descriptions of this papillary surface architecture, particularly on MRI, have been limited. In the present case, the papillary configuration of the tumor surface was clearly demonstrated on contrast-enhanced MRI, and radiologic-pathologic correlation confirmed that this imaging finding corresponded closely to the underlying tissue morphology. Recognition of papillary surface architecture on contrast-enhanced MRI may therefore provide a useful diagnostic clue that supports consideration of adenofibroma in the differential diagnosis of endometrial cavity tumors.

The present case revealed two distinctive imaging findings: linear enhancement along the tumor surface in the early phase and a papillary surface appearance with progressively increasing enhancement in the delayed phase. Extensive squamous metaplasia, an uncommon histological feature in uterine adenofibromas, likely contributed to the observed imaging features.

## Conclusions

This report highlights the diagnostic importance of papillary surface enhancement patterns on MRI in the evaluation of tumors within the endometrial cavity. Identifying these features may help distinguish uterine adenofibroma from malignant mixed epithelial-mesenchymal tumors. To the best of our knowledge, based on a literature search of PubMed and Google Scholar using the terms “uterine adenofibroma,” “MRI,” and “papillary enhancement,” this enhancement pattern has not been specifically described as progressive delayed papillary surface enhancement on dynamic MRI. Nevertheless, additional cases are needed to confirm this finding. Further accumulation of cases with radiologic-pathologic correlation is necessary to enhance diagnostic accuracy for this rare entity.
